# Towards capturing meaningful outcomes for people with dementia in psychosocial intervention research: A pan‐European consultation

**DOI:** 10.1111/hex.12799

**Published:** 2018-06-19

**Authors:** Laila Øksnebjerg, Ana Diaz‐Ponce, Dianne Gove, Esme Moniz‐Cook, Gail Mountain, Rabih Chattat, Bob Woods

**Affiliations:** ^1^ Department of Neurology Danish Dementia Research Centre Rigshospitalet University of Copenhagen Copenhagen Denmark; ^2^ Alzheimer Europe Luxembourg Luxembourg; ^3^ School of Health and Social Work Faculty of Health Sciences University of Hull Hull UK; ^4^ Centre for Applied Dementia Studies University of Bradford Bradford UK; ^5^ Dipartimento di Psicologia University of Bologna Bologna Italy; ^6^ Dementia Services Development Centre Wales Bangor University Bangor UK

**Keywords:** consultation, dementia, methods, outcome measures, patient and public involvement, positive psychology, psychosocial, quality of life, social health

## Abstract

**Background:**

People with dementia are often marginalized and excluded from influence, also in relation to dementia research. There is, however, a growing requirement for inclusion through Patient and Public Involvement (PPI), but there is still limited knowledge on how researchers can fully benefit from the involvement of people with dementia in the development and testing of psychosocial interventions. This paper describes the results of a pan‐European consultation with people with dementia, synthesizing their views on outcomes of psychosocial interventions.

**Objective:**

To involve people with dementia in establishing what are meaningful outcomes when participating in psychosocial interventions.

**Setting and participants:**

Consultations took place at four divergent sites across Europe, involving twenty‐five people with dementia from nine European countries.

**Methods:**

The methods used for the consultation were developed through an iterative process involving people with dementia. Data from the consultation were analysed from a thematic analysis approach.

**Results:**

The results suggested that people with dementia wish to participate in interventions that enhance their well‐being, confidence, health, social participation and human rights. This highlights a need for improvements in psychosocial research to capture these outcomes.

**Discussion and conclusions:**

Involving people with dementia in discussions of psychosocial interventions has enhanced our understanding about meaningful outcome measures in research and methods of data collection. This study suggests that new outcome measures in psychosocial research are needed where concepts of positive psychology and social health can guide innovation and outcome measurement.

## 1 INTRODUCTION

Living with dementia often leads to marginalization by society. People with dementia are not included and adequately involved, even with issues directly related to their life situation. For instance, people with dementia are often not informed about their diagnosis and treatment,^1^ and when conducting research, or developing new health or social services, the opinions and experiences of people with dementia are seldom requested or shared.^2^ Nonetheless, a shift in paradigm is evolving, giving people with dementia more opportunities to influence policies, practices and research. This shift is reflected in increased political awareness, for example giving rise to National Dementia Strategies^3^ and emphases on dementia‐friendly societies in many countries.^4^ The shift has come about as a result of several initiatives, particularly the effort of individuals sharing their views and experiences of living with dementia and the united effort of stakeholders in NGOs.^5^ There is also a growing emphasis on Patient and Public Involvement (PPI) when conducting research, but this is usually with respect to recruitment to studies^6^ and not direct inclusion in the research process.^7^

To fully benefit from the involvement of people with dementia we need knowledge on how this might be best accomplished. Alzheimer Europe, together with INTERDEM and the European Working Group of People with dementia, has published a position paper on the involvement of people with dementia in research through PPI, reflecting on the benefits and possible challenges associated with this.^8^

Aligned to this, the overall objective of the consultation described in this paper was to have people with dementia involved in the development of meaningful outcome measures within the field of psychosocial intervention research.

Evidence is emerging for psychosocial intervention in dementia,^9^, ^10^ but sound methodology about outcome measures remains important.^11^ Traditionally, outcome measures in dementia research have focused on cognition, functional ability and symptoms that demonstrate the gravity of the loss/deficit paradigm in dementia care,^11^ but there is a need for outcome measures which truly mirror meaningful outcomes for people with dementia. The concepts of well‐being and “living well” with dementia are essential in many psychosocial interventions,^12^, ^13^ but rarely genuinely captured and reflected in outcome measures. Well‐being and “living well” with dementia is related to positive psychology, a theoretical framework and approach which is emerging in relation to in dementia.^14^, ^15^ Its focus on positive characteristics and capacities of individuals to enable well‐being and “living well” can also be linked to the understanding of health as a positive construct.^16^ A reformulation of the WHO definition of health has been proposed which moves towards a more dynamic definition, based on the ability to physically, mentally and socially adapt and self‐manage.^17^ Within this framework, social health is characterized by having the capacity to fulfil one’s potential and obligations, the ability to manage life with some degree of independence, despite a medical condition, and to participate in social activities.^17^ This concept of social health has been operationalized within the field of dementia in a European consensus paper,^18^ encompassing well‐being at multiple levels, and emphasizing social health as a favourable framework within which to focus on positive outcome measures in dementia research. We need to adopt these new understandings of the relationship between well‐being and health when living with dementia and incorporate them into the field of psychosocial research.

## 2 RESEARCH AIM AND QUESTIONS

This study aimed to involve people with dementia in establishing what are meaningful outcomes when participating in psychosocial interventions. We also wished to increase our knowledge of how to involve people with dementia in research consultations and fully benefit from their participation.

To accomplish the project aims we formulated the following research questions:

Opinions and experiences of people with dementia:

1. What psychosocial interventions do people with dementia consider meaningful?
2. What do people with dementia consider to be meaningful approaches to capture the essence of psychosocial interventions through research?

Methodological issues:

- How can we consult people who are living with dementia in diverse circumstances about the development of new psychosocial interventions and research related to this?

## 3 METHODS

The consultation presented in this paper was part of a larger study,^19^, ^20^ where the overall aim was to chart new territory in psychosocial interventions outcome research.

### 3.1 Design

The methods and material used for the consultation were developed through an iterative process involving people with dementia. An exploratory consultation in relation to the design of this study was conducted in the UK. It involved five people with dementia and four family caregivers from a group established to advise researchers on ideas and proposals for dementia research. This consultation identified a number of important factors for successful consultation with a group of people with dementia, such as the importance of an appropriate environment and setting, the need for familiarity with the setting, enhanced facilitation and, in some cases, the need for support for communication.

The results of this exploratory consultation were discussed at a workshop with 13 dementia experts from the pan‐European Interdem network who had a special interest and expertise in the subjects of this study.

Based on this workshop, materials for the consultation were developed: an information sheet for participants and a guide for moderators of the group. The guide for moderators served as a common framework for conducting the consultation. It suggested topics and questions designed to uncover issues related to the key aims and objectives of the project. Also a vignette (a short imaginary situation) was created to be used during the consultations to facilitate discussion^21^ (see Appendix S1). The material was developed in English, and later translated, and minor adaptions were made to fit local culture and terminology in the countries participating.

The process of preparing and conducting the consultation, and the data analyses, took place during 2015. Afterwards, two workshops took place during 2016. First, 26 dementia experts from the Interdem network, who had a special interest and expertise in the subject, met to debate the initial findings, and later, the authors met to shape the final results.

### 3.2 Settings

The consultation took place at four sites in Europe. Groups had 3‐9 participants, and they were brought together through a mix of purposeful and convenience sampling. This made it possible to have representatives with a common experience of living with dementia, but from a range of backgrounds, nationalities, ages and different types and stages of dementia, thereby giving a voice to people with different experiences, views and preferences.

One consultation was conducted in Belgium with a multinational group of people with dementia who were part of an on‐going advisory group facilitated by Alzheimer Europe. The three other consultations were conducted at an Alzheimer’s Society office (UK), a meeting place used for group activities for people with dementia (Italy) and a memory clinic running post‐diagnostic support programmes (Denmark). The settings and the professionals attending were familiar to the participants. In some cases, the participants were also familiar with each other, for example attending the same group activity.

The overall study was designed to have separate consultations with groups of people with dementia and family caregivers respectively. This paper includes the results of consultations with people with dementia. In the UK consultation, however, the groups decided not to split; all participants clearly indicated that they were comfortable with participating and giving their opinion in a joint group. The results of this consultation were carefully split during data analysis, and this paper only includes data generated from people with dementia.

In the Alzheimer Europe group, three participants with slightly more advanced dementia had communication support from caregivers during the consultation, for example help to explain questions or elaborate responses.

The consultations ran for between 1 and 1.5 hours and were facilitated by one or two researchers at each site. At some sites, researchers were supported by additional staff.

In full agreement with all participants, the consultations were audio‐recorded.

### 3.3 Participants

Twenty‐five people with dementia from nine European countries participated in the group consultations. Participants were between 49 and 81 years old, with a majority of people being over the age of 65. There were 14 women and 11 men, who were mildly to moderately affected by dementia. The characteristics of the participants and groups are presented in Table 1.

**Table 1:** Details on consulted groups, participants and consultations

Groups and participants				Consultations			
Group location	Setting	Group characteristics	Participant characteristics^a^	Moderator(s)	Duration	People with dementia or mixed group	Support for people with dementia
Alzheimer Europe	Hotel meeting facility in Brussels	Members of the European Working Group of People with dementia^b^Participants were familiar with each other, except 1 new member	9 people with dementiaAge: 56‐71M/F: 3/6	Psychologist/PhDSocial Worker/PhDBoth familiar to the group/participants	1¼ h	People with dementia	3 family caregivers were present to support 3 group membersFlip chart record of the discussion and printed handouts
UK	Local Alzheimer’s Society offices	Members of the SHINDIG group^c^Participants were familiar with each other	4 people with dementiaAge: 1: 61, 3 > 65M/F: 3/13 caregivers (all spouses)	Lead researcherSenior clinical nursePhD studentAll familiar to the group/participants	1½ h	Mixed group of people with dementia and family caregivers	Flip chart record of the discussion
Italy	Local Alzheimer’s Society offices	Members of a cognitive stimulation groupParticipants were familiar with each other	7 people with dementiaAge: 72‐79 yM/F: 3/4	3 psychologists2 other professionalsAll familiar to the group/participants	1 h	People with dementia	None
Denmark	Memory clinic	Members of a group support programme for people with dementiaParticipants were familiar with each other	5 people with dementiaAge: 49‐81 yM/F: 3/2	1 PsychologistFamiliar to the group/participants	1 h	People with dementia	Framework of the consultation presented on PowerPoint slidesFlip chart record of the discussion

All participants were diagnosed with dementia and were living with dementia at a mild‐moderate stage.

a The level of details differs due to the nature of the different group settings.

b The EWGPWD is comprised of people with dementia. They work to ensure that the activities, projects and meetings of Alzheimer Europe duly reflect the priorities and views of people with dementia. The group operates independently, with its own Board and agenda of activities. Nationalities of members: Germany, Czech Republic, England, Scotland, Ireland, Jersey, Norway, Finland and Slovenia.

c The SHINDIG group is a citywide forum which provides opportunities for those living with dementia to give views and opinions on local services and developments.

### 3.4 Procedure

Consultations were facilitated by researcher AD and DG in the Alzheimer Europe setting, researcher GM in the UK, researcher RC in Italy and researcher LØ in Denmark. The consultation was based on a focus group approach. The facilitators initiated the consultation by giving a short introduction of the general topic. Next, the specific topics from the interview guide were introduced stepwise, supported by the short vignette to facilitate discussion. Topics and questions were addressed in a flexible way and adapted to the pace of the group. Some participants found it difficult to relate to the general topics and challenging to go from the vignettes to more specific personal opinions and discussions. In these cases, participants were encouraged to think about their personal experience of an activity they knew, in order to give them the opportunity to relate to the topics in a more personally meaningful and concrete manner.

### 3.5 Data analysis

Each audio‐recorded consultation was separately transcribed. Subsequently, the data were processed and interpreted based on the principles of the framework approach.^22^

As part of this process, a coding framework was created through an iterative process between the researchers who had facilitated the consultations. To set out a preliminary common framework for analysing the consultations, the Alzheimer Europe consultation was used as benchmark, and two of the researchers (AD and DG) carried out an open coding on the transcription of this consultation. Secondly, these codes were used to develop a common framework of predetermined categories, which was discussed and adapted by all researchers involved in the consultations. Thirdly, these categories were used as a common framework to code all consultations. Coding was, however, not restricted to these predetermined categories of the common coding framework. Researchers were free to add new codes and categories if necessary, in order to capture the varied experiences and views represented by all participants, and to avoid neglecting important details.

The initial coding process was performed in the original language, and at the final stages, the essential parts of the transcripts from the Italian and Danish consultations were translated into English.

The results of the four consultations were discussed and processed by the researchers at two workshops, first to synthesize initial findings and then to shape the definitive conclusions.

## 4 RESULTS

The research aims and questions of this study are used as a framework to summarize the results from the consultation. The research questions serve as headlines and are followed by subheadings of themes that emerged from the consultations. Key constructs summarize the subsequent narratives and related quotations.

### 4.1 Research question: What psychosocial interventions do people with dementia consider meaningful?

#### 4.1.1 Theme: Individual needs and rights

##### Key constructs

Preconditions for participating, accepting risk, equal rights, individual needs and well‐defined activities.

##### Narrative

Participants mentioned several requirements that should be met before they would consider taking part in any activities or research. These included feeling safe and that their needs were understood by a professional who has a sufficient understanding of dementia.

Before I would even consider attending any sessions…I would need to feel that I was going to be in a safe, secure place, where people would know what my needs were…, rather than as I’ve had in the past. I turn up to do something, looking forward to it, and to find out that people don’t even understand dementia.

It would be a bad experience for me if something was too overwhelming…if there were too many.

While feeling safe was mentioned, the right to take risks was also emphasized. Some participants were concerned that people with dementia were sometimes denied the right to do activities where they could risk injury.

At the core of the preconditions for taking part was the desire to be treated like a human being who wants to feel good and enjoy life rather than as an “animal,” “object” or “specimen under a microscope.”

Some participants also emphasized that they expected specific information on the interventions they were invited to participate in, in order to decide if it was the right thing for them. “One would also like to know how it works…for instance if it makes your better at remembering or something.”

#### 4.1.2 Theme: Social participation

##### Key constructs

Social engagement, socializing, social inclusion, dignity, reciprocity, sharing and tailored activities.

##### Narrative

The importance of socializing with other people and social engagement was described in the context of a desire for companionship and understanding from others. The joy of engaging in social relations, both familiar and new, was illustrated in different ways.

Examples partly reflected the need to feel included in social contexts. However, issues of reciprocity and social dignity were also identifiable in the discussions. For example, participants emphasized the importance of being an equal member of a group, “being part of everything that’s not dementia” and not being treated as special, but nevertheless having appropriate attention paid to their needs; and also the importance of activities which help to overcome the feeling that “I am useless” and “a lesser person” in relation to others. Beliefs about inherent dignity were reflected in a plea to researchers to treat people with dementia as human beings who like to feel good. As one participant stated: “I am not an animal, I am a human being…I don’t want to feel like an object.”

The issue of reciprocity was raised several times in relation to individuals, groups, the community and researchers. Participants described their perception of receiving something which they enjoy or appreciate (eg, yoga classes or walking with other people) and giving something back by educating people about dementia, contributing towards changing perceptions of dementia and contributing towards research efforts.

The importance of having access to activities that are tailor‐made for people with dementia was also discussed. Activities that gave them an opportunity to meet other people in a similar situation, sharing their situation and enjoying shared activities and interests.

And the beauty of this place is…the various things that happen, it fills an empty diary, but it also produces friendly people every time, supportive friendly people.

I can talk here, everyone can have his say, I like it.

We are a bit boring aren’t we in life…and it would be nice to get together with people like, you know, a group of us sort of thing. Because when we are all together and mix…, we all understand each other, so in a similar group altogether—it would be ok.

#### 4.1.3 Theme: Confidence, positive emotions and sense of competence

##### Key constructs

Enjoyment, feeling good, reaching or touching emotions, creation, mastery and achievement.

##### Narrative

A recurring issue throughout the consultations was that people with dementia want to enjoy themselves, have fun and feel good. Participants expressed interest in activities, programmes or forms of support which help them to do so, and the desire to judge the success of such interventions mainly on the basis of whether they result in enjoyment, having fun and feeling good.

Several references were made to certain activities/interventions that touch a person’s emotions (eg, music or arts) and enable the person to access emotional memories which would otherwise not have come to the surface, creating an emotional reaction. One person emphasized that it was not about triggering factual memories, but about emotional memory and emotional happiness.

Many examples were given of activities that the participants were able to do, and how being able to do them meant much in terms of personal satisfaction, the sense of being able to master something and having a sense of control. One person said that joining the group activity for people with dementia gave him the opportunity to be completely independent for a while, autonomous and free to express his point of view.

#### 4.1.4 Theme: Health

##### Key constructs

Cognition, functional ability, mental health and physical health.

##### Narrative

Participants varied in their opinions on whether interventions should be applied to try to make a difference to their cognitive and functional ability. Some wanted specific personalized ways of coping with memory problems and to be able to participate in daily activities, and they mainly emphasized a wish to maintain ability rather than improve it.

I don’t want to lose memory, sometimes I have something like memory loss, and I think that interventions like this one can be useful for my memory.

The importance of doing something because it affects physical and mental health was also highlighted.

For example, I was always anxious before I joined the group; I always had the need to check my purse looking for something. Now I don’t do it anymore, the intervention reduced my tension and I really appreciate that.

I joined a walking group…. I find it twofold, one that I find myself feeling very well, after I walk seven or eight kilometres…and so I feel that this has an impact on my life because I am forced to talk to a lot of people.

Specific physical health‐related outcomes included improving balance, having a strong core, preventing falls and remaining mobile.

#### 4.1.5 Theme: Well‐being

##### Key constructs

Coping, sense of control, confidence, identity, self‐esteem, quality of life and well‐being.

##### Narrative

Participants emphasized that coping with dementia did not necessarily mean effectively managing various everyday situations, but rather about feeling OK about having dementia, being emotionally able to deal with any difficulties which may arise, and focusing on what was still possible. Some participants spoke with great enthusiasm about activities which gave them a sense of control and a feeling of confidence as a result of being able to achieve or create something (eg, training dogs, doing woodwork, playing an instrument or singing). Being able to produce or create something was linked to self‐esteem and self‐respect.

To give me a sense of ‘me’ again, a sense of identity.

They want me to be part of the group, not because I have the illness, but because I am [xx].

Just feeling well, at peace with yourself, perhaps for a while not dwelling on problems, and you get some period of comfort. It is just joy. And I love it.

In general, participants took it for granted that problems occurred in daily life. Quality of life and well‐being seemed to be a reflection of “getting on with life” despite problems that might arise. Some participants referred to living well with dementia in the context of doing something which makes them feel good and happy, being part of the wider social group, not dwelling on problems, but getting on with life and coping with setbacks and difficulties.

…just feeling well at peace with yourself, perhaps for a while not dwelling on problems and you get some period of comfort. I get absolute joy from walking the dog in woods, just being back with nature.

Sometimes I need a ‘change of scenery’—I leave behind my family for a while and all my worries.

### 4.2 Research question: What do people with dementia consider to be meaningful approaches to capture the essence of psychosocial interventions through research?

#### 4.2.1 Theme: Views on evaluation and outcomes

##### Key constructs

Momentary versus long‐term outcomes, authenticity, accuracy of feedback, emphasis on predominantly qualitative methods and resistance to self‐report instruments and questionnaires.

##### Narrative

The consultations revealed various views and opinions on what would be the most ideal way to capture the essence of interventions and how to evaluate them.

Some participants found that recording outcomes and reactions in the moment, both during activities and just at the end of them, should be the main focus. Various ways of doing this were proposed such as interviewing, filming or audio recording, observing body language and expression and taking note of reactions.

“In the moment” evaluations were described as being most authentic and most accurately reflecting what people actually think and feel, and therefore easier for people with dementia to manage.

I am worried that I wouldn’t remember many things if researchers would ask me questions some weeks after the intervention.

I think ‘in the moment’ is the most honest you’re gonna get… .I think filming is a good way because…we would forget what you filmed—as everybody does—and you get the real reactions.

Another commented that what is measured “in the moment,” such as happiness, might also have a lingering effect and is just the trigger, suggesting that long‐term effects should also be measured.

Potential difficulties and complex issue of obtaining “in the moment” feedback was also discussed. Difficulties included social desirability (eg, wanting to please, just saying what the researchers might want to hear), being too tired to respond, or being put on the spot and feeling under pressure to respond. On the other hand, they also recognized the problem of being asked later and having forgotten how they felt about the intervention. It was acknowledged that the same method for obtaining feedback might not be suited to everyone across all disabilities due to dementia.

The importance of getting information and observation from other people (eg, family caregivers) was discussed. Some found it particularly important if changes over a period of time are to be measured. One participant suggested: “You could also ask the relatives. I have experienced that you get a lot of input from them. Sometimes it is hard to see the little changes. You don’t see it yourself.”

Some participants were very critical of questionnaires.

If you talk with someone, you can really share your thoughts, your idea. I don’t think that word and pencil questionnaires can grasp what you really think.

Participants in general agreed that people with dementia should be more involved in the preparation of interventions to ensure that they correspond to their individual needs and preferences.

It was also suggested that users should be involved in the analysis and interpretation of observations and measurements, for example. video recordings, to make sure that the analysis came to the right conclusions.

## 5 DISCUSSION

### 5.1 Main findings

The consultation was conducted to embody the voices of people with dementia in understanding what they consider to be meaningful aspects of psychosocial interventions, including the identification of outcome measures that truly mirror the benefits and possible drawbacks of these interventions.

An essential aim of the project was to include a broad variety of people living with dementia, and to present all their views. The consultation took part at four sites across Europe, with participants representing various experiences of living with dementia, and researchers found a number of common themes among participants.

In general, the findings showed that the people with dementia who were consulted favoured psychosocial interventions that imply social engagement and inclusion, and that they wanted to be involved in settings and activities where they sensed respect for dignity and reciprocity. Various views on what they found important to gain from interventions and activities were presented. Activities that imply enjoyment and reaching or touching emotions were in general emphasized, including activities that give a sense of mastery and achievement. In this way, some activities and accomplishments seem to have a symbolic function linked to self‐esteem and identity, rather than being simply ways to pass time or keep active.

These themes strongly relate to the concept of social health, as defined by Huber et al^17^ and operationalized in relation to people with dementia by Droes et al,^18^ and to a positive psychology approach to living with dementia.^15^ These new concepts are essential in the future development of meaningful interventions within the field of psychosocial interventions in dementia.

Understanding the contextual influents on participant reports is an important consideration in psychosocial research.^23^ When discussing actual outcomes, participants had varying views on the importance of outcome measures on cognition and ability, perhaps reflecting differing contexts of the four study sites.^24^ Some questioned the relevance of cognitive and functional ability to their overall quality of life. Those in favour of outcome measurements that address cognition and ability stressed the importance of emphasizing the maintenance of function rather than improvement. Mental health was described as a positive outcome measure that was generally sought. In particular, having confidence and a sense of control was emphasized. This is in line with the lack of convincing evidence that the level of cognitive impairment is associated with lower quality of life, whereas depression is consistently associated with decreased quality of life.^25^ It also underlines how other factors such as choice, self‐determination and maintaining independence influence the quality of life of people with dementia.^26^, ^27^

These results are also consistent with the concepts of social health and positive psychology, and they underline the need to develop new outcomes that reflect these positive constructs.

Participants also stressed the importance of activities to meet the individual and various special needs of people with dementia. There were, however, various opinions on this, underlining the need to delicately balance the need for activities and settings where people with dementia feel safe, with their right to accept risks and have equal access to activities that are meaningful and rewarding at a personal level. Such reflections reinforce a continued discussion on human rights and ethics related to dementia when designing and applying psychosocial interventions.

When discussing the actual application of outcome measures both momentary and long‐term outcomes were mentioned, and there was a preference for momentary outcomes bringing more authenticity and accuracy to the feedback of people with dementia. There was also an emphasis on qualitative methods. These views underline the importance of using mixed methods when assessing the outcomes of psychosocial interventions in dementia.^28^

The study also addressed the methodological issues of how to consult people living with dementia under different conditions and from various backgrounds and have them contribute. The design of this study gave access to the experiences and opinions from a broad spectrum of people living with dementia. Through an iterative process, involving people with dementia, we succeeded in developing a structured methodology that could be applied in an international setting with a varied group of people with dementia. This structured framework gave us the opportunity to gather rich and diverse data, which could be condensed to answer the research questions that we set out to address in this study.

### 5.2 Limitations

Despite the broad recruitment of participants for this study there are still limitations with regard to the variety of people with dementia represented in the study. For instance, people with dementia who do not take part in psychosocial activities or advisory groups were not represented among participants. Also, people with severe dementia were not included in this study. These limitations could have implications for the generalizability of results. If people with severe dementia had been included a different design would have been needed and special efforts made to elicit their views.^29^, ^30^

Another limitation to this study is the variation of caregiver involvement at the four sites. As part of the overall project, caregivers were invited to participate in a separate consultation addressing their involvement in and views on psychosocial activities. The results from this consultation form a separate study. However, at one of the settings (UK) caregivers took part in the consultation together with people with dementia, as people with dementia and caregivers decided not to split into separate groups. Caregivers were also present to support communication for three of the participants in the Alzheimer Europe working group consultation. They were not directly involved in the discussion. Still, in both situations, it is possible that the mere presence of caregivers may have influenced the results of the consultation. On the other hand, having a trusted person provide support to a person with dementia can be facilitating and empower a person with more advanced dementia to follow the discussion and contribute. This discussion underlines the importance of achieving the appropriate balance between carers supporting people with dementia to have a voice, and carers speaking for the person with dementia and inhibiting the person’s own contribution.

A further limitation is that people with dementia were not directly involved in the processing or interpretation of results of this consultation. It can seem paradoxical, as such involvement was requested by some participants in the consultation. We acknowledge that this should have been considered as part of the original design of the study, but for practical reasons, it was not possible to change the design during the study.

In the light of the above limitations, we recommended that future research also involves people who are not already participating in interventions. We acknowledge the need to include people with a more severe degree of dementia. Future studies need to be designed to meet the special needs and ethical considerations related to their participation. We also recommend that future research explores how consultations with people with dementia can be fully coprocessed and endorsed by people with dementia.

These considerations are further elaborated and discussed in Alzheimer Europe’s position paper on PPI (patient and public involvement)^8^ which is related to the current study with regard to collaboration and scope of interest. The position paper outlines the potential challenges, risks and benefits of the much needed and meaningful involvement of people with dementia in research, ranging from consulting people with dementia at various stages of research to more comprehensive involvement in research.

## 6 CONCLUSION

The results of this study clearly underline benefits of involving people with dementia when planning and conducting psychosocial interventions research. The views expressed by people with dementia support the position that in addition to consideration about maintaining health in a broad sense, activities and interventions could focus on enhancing dignity through social engagement, and reciprocal contribution to society, and should take into consideration individual needs, preferences and rights. These values are embedded in concepts of social health and positive psychology, which are emerging within the field of psychosocial interventions for people with dementia.

This consultation also demonstrates that given the opportunity and taking into account special needs, people living with dementia within a variety of settings across Europe can contribute to research on psychosocial interventions in dementia.

## CONFLICT OF INTEREST

None.
