# Supplementary material for: Towards capturing meaningful outcomes for people with dementia in psychosocial intervention research: A pan‐European consultation
Source: Health Expect. 2018 Jun 19;21(6):1056–65. doi: 10.1111/hex.12799 (PMC6250864; doi:10.1111/hex.12799)
Supplement: Supplementary file 1 [file HEX-21-1056-s001.docx]

**Appendix 1: Vignette and suggested themes and questions for the group consultation**

**Vignette about people with dementia participating in a research project:**

*Imagine…in connection with you having dementia, you have been offered the possibility to attend a series of ‘singing and remembering’ sessions…. Please try to imagine the impact that such sessions might have on your life, health, condition or wellbeing (both at the time and afterwards).*

*Please also think about how you would personally assess whether or not they were successful or beneficial.*

It was pointed out to researchers that this was just an example. They were free to use other examples of interventions that might include exercise routines, cognitive stimulation etc.

**Suggested themes and questions:**

- In connection with the series of ‘singing and remembering’ sessions, what impact do you think they might have on your quality of life, health/condition or social contact with others?
- Which aspect of having/living with dementia would you like to see improved as a result of taking part in a research activity, programme or intervention? In other words: if you took part in a session, in which areas of your life would you hope to see an improvement?
- On what basis (or according to which criteria) do you think researchers should assess whether or not a particular programme or intervention was successful/effective?

It was pointed out that researchers were free to split, reformulate, adapt, and to add questions and emerging themes.
